# “Snip, snip, cure”? Philosophical, legal and biomedical perspectives on novel somatic genomic therapies

**DOI:** 10.1007/s11019-025-10284-5

**Published:** 2025-08-11

**Authors:** Johanna Risse, Lothar Pietrek, Tobias Cantz, Merlin Krzemien, Jan Schnalke, Reto Eggenschwiler, Thomas Heinemann, Hans-Georg Dederer

**Affiliations:** 1 Institute for Medical Humanities, Medical Faculty, University of Bonn, Venusberg-Campus 1, 53127 Bonn, Germany; 2 Chair of Constitutional and Administrative Law, Public International Law, European and International Economic Law, Faculty of Law, University of Passau, Innstraße 39, 94032 Passau, Germany; 3 Translational Hepatology and Stem Cell Biology, Department of Gastroenterology, Hepatology, Infectious Diseases, and Endocrinology, Hannover Medical School, Carl- Neuberg-Str. 1, 30625 Hannover, Germany

**Keywords:** CRISPR/Cas, Genome editing, Gene therapy, Postgenomic era, Advanced therapy medicinal products

## Abstract

The advent of innovative techniques, such as the CRISPR/Cas system, has opened up a new range of possibilities for modifying the genome, with the potential to address previously unmet therapeutic needs of patients with genetic diseases. These new possibilities have not only raised ethical concerns but also challenged existing classifications of genome modification techniques. While the legal status of some of these new therapies remains uncertain, there is an ongoing debate within philosophy of biology about the information-related metaphors adopted by scientists to describe and classify the genome and its therapeutic modification. Given the continuing advance of new genomic therapies, we show, employing an interdisciplinary approach, that a comprehensive framework for the classification of these technologies is needed to resolve legal and philosophical issues. The first section provides an analysis of the current state of novel genome-modifying techniques in medical genetics. In the second section, we assess the regulatory status of these techniques within the European regulatory framework for advanced therapy medicinal products (ATMPs). Drawing on these results, we argue in the third section from a philosophical perspective that metaphors, such as ‘editing’ the genome, which are based on a conception of the genome as linear information, cannot adequately capture the breadth of advanced genomic technologies. To accurately categorise these techniques in a manner that meets their diverse applications, we propose introducing the umbrella term ‘somatic genomic therapies’ (SGTs). Urging an integrative approach to defining and classifying new technologies in medical genetics, we advocate for the development of an integrative concept of SGTs.

## Introduction

With the emergence of a new generation of genomic technologies, medical genetics is on the way to a new era in which genomic and cell-based therapies will transform our ability to prevent, treat, and cure diseases. While the discourse around genetics has always been shaped by powerful narratives and metaphors, the discovery of CRISPR/Cas (Clustered Regularly Interspaced Short Palindromic Repeats/CRISPR associated) in particular has intensified this tendency. Journalists and scientists alike revel in suggestive headlines such as “Genetic scissors: a tool to rewrite the code of life” (The Nobel Committee for Chemistry 2020), “Snip, snip, cure: correcting defects in the genetic blueprint” (University of Cambridge 2017), or “In gene editing, the genetic code now has a workable text editor. Will this mean Ctrl + X for disease and Ctrl + V for talent?” (Parrington 2015). These metaphors vary in how they present the novel techniques in question (e.g. scissors, toolkit, text editor), in what the techniques do (e.g. editing, targeting, rewriting), and in how they understand the genome as their underlying target (e.g. code of life, book of life, blueprint, text, letters) (Nelson et al. 2015). What these metaphors have in common is that they draw on a notion of the genome as linear information. Although such imagery may have been a helpful conceptual tool in earlier phases of the development of molecular biology, its applicability to new technologies such as CRISPR/Cas, which can be applied at different functional genomic levels, needs to be called into question (The Nuffield Council on Bioethics 2016; Nelson et al. 2015; Perrault and O’Keefe 2019; O’Keefe et al. 2015). This is due to the fact that the body of metaphors of information used understates the advancements and complexities of contemporary genomic research, particularly in relation to the mechanisms underlying diseases. Eventually, these metaphors imply inappropriate notions of genomic therapies because they downplay and obscure the complexity of manipulating the genome.

In this paper, we argue that recent advances in genomic research require a revision of definitions and classifications concerning the genome and thus genomic therapies. As will be shown below, such therapies can be administered by very heterogeneous means, leading to the ever-growing challenge of creating a clear classification system that captures their key characteristics for a revised normative framework. This enterprise is linked to a “deep-philosophical line-drawing problem”^1^: as legal scholar Sherkow et al. (2018) suggest, all therapies, including small-molecule drugs, may be considered to affect a patient’s genome in some way. For this reason, it is necessary to address the question of what distinguishes genomic from non-genomic therapies. The analysis of the demarcation problem between genomic therapies and other treatments has received little attention so far. This poses a problem because the classification of therapies as genomic therapies may have far-reaching ethical and regulatory consequences (Sherkow 2018; Nicol 2017). For instance, transcriptomic and epigenetic approaches such as mRNA-based therapies or CRISPRi can achieve therapeutic modulation of gene expression without altering the genomic DNA itself. Some of these interventions occur entirely within the cytoplasm of the cell and do not require access to the nucleus. Therefore, we introduce somatic genomic therapy (SGT) as an umbrella term that retains the inclusion of classical gene transfer techniques, but is not confined to them. Instead, it encompasses all therapeutic interventions that make use of the tools of functional genomics, i.e. tools that, following postgenomic definitions, act on functional elements of the genome, transcriptome or epigenome to influence gene expression. These include not only DNA-level edits, but also targeted regulation of transcription, translation, or chromatin states.

We address the classification of these emerging techniques by employing an interdisciplinary approach: First, in order to illustrate recent developments, we depict the broad biomedical spectrum of novel genomic techniques, showing how they interfere with quite different levels of genomic functions and thus differ from each other in the mode and place of action within the cell as well as the type of molecules used, e.g. nucleic acids. Thus, it becomes evident that the multitude of novel techniques cannot be categorised within the narrow framework of conventional gene therapy, which works by adding a functional copy of the gene, predominantly via viral vectors. Nor can they be accurately represented by a simple metaphor. Second, against this background, we perform a legal case study with the aim of illustrating the practical difficulties that arise regarding SGTs within the context of the EU regulatory framework for advanced therapy medicinal products (ATMPs). This analysis reveals that applying this legal framework to SGTs leads to inconsistent results in some cases. Seemingly, these inconsistencies are caused by metaphors and paradigms, which most likely have inspired the EU legislature. As we show, the current legal definition of Gene Therapy Medicinal Products (GTMPs), which is an ATMP subcategory, is based on the conception of the genome as linear information and, thus, presupposes GTMPs to be medicinal products that perform a gene transfer. Moreover, the EU legislature has initiated a legislative procedure (EC 2023a) aimed at revising this definition. However, also the revision relies on a metaphor, namely on the metaphor of “genome editing”, which implies again a linear and textual understanding of the human genome. Since this metaphor does not reflect current scientific understanding of the genome as a non-linear and multilayered system, the proposed revision of the GTMP definition gives rise to regulatory uncertainty for SGTs as well. Third, in order to substantiate a philosophical justification for the proposed classification, an investigation is conducted into the ongoing debate in the so-called postgenomic era regarding the conceptual issues surrounding the genome and genomic therapies. In this section, we demonstrate the conceptual shortcomings of the metaphor of ‘editing’ the genome as it is based on an understanding of the genome as static and linear information and fails to integrate ‘postgenomic’ research. Therefore, we argue that a dynamic and multidimensional view of the genome should provide the foundational concept for SGTs. Subsequently, we develop the outlines of an integrative ‘postgenomic’ concept that also addresses the problem of demarcation between genomic and non-genomic therapies.

## Biomedical perspective: gene therapy, genome editing, and beyond

The idea of gene therapy as a potential therapeutic approach for human genetic disorders was introduced by Theodore Friedmann and Richard Roblin (1972) in 1972. The subsequent years saw the development of what are now considered conventional gene therapies, which aim to restore the function of the mutated gene by introducing an additional functional copy of this gene into cells. However, it was not until much later that ‘genome editing’, which potentially enables the precise modification of any genomic loci, became a viable possibility. In 2005, the first study to use zinc finger nucleases for modifying DNA in human cells was published (Urnov et al. 2005). The study coined the term ‘genome editing’ to describe a method that is distinct from gene therapy, drawing on “an analogy with word processing (‘clicking’ on a stretch of genomic text to change it; keyboard-like precision control over the outcome)” (Urnov et al. 2018). The advent of CRISPR/Cas has led to a notable increase in the utilisation of ‘genome editing’ within the scientific laboratory setting, largely due to its affordability, rapid implementation, and user-friendly design. As a result, the term ‘editing’ is now commonly used to herald a new era of disease cures (Doudna 2020). While the metaphor of ‘editing’ is deeply embedded in scientific and public discourse, ethicists are critical of its evaluative connotations, which they argue obscure the complexity of genome manipulation (The Nuffield Council on Bioethics 2016; Nelson et al. 2015; Perrault and O’Keefe 2019; O’Keefe et al. 2015). The term ‘genome editing’ suggests a rather simple procedure, but in practice, genome therapies are a highly complex form of medicine. The main challenge faced by both conventional and novel approaches is the safe and effective delivery of therapeutic molecules to targeted tissues or cells, for which often tailored delivery vehicles are used, which are known as vectors (Taha 2022). Each vector system is distinctive in terms of the safety and delivery issues it presents, including the risk of unintended genome alterations. These so-called off-target effects have the potential to cause serious side effects, such as the activation of oncogenes or the inactivation of tumour suppressor genes, which may culminate in the development of cancer (Khoshandam et al. 2024). In order to evaluate the risks associated with a therapy, it is essential to recognise the distinct modes of action involved (EMA 2018). As a result, the mode of action is an important discriminator in the development of ethical and regulatory frameworks. Another important discriminator is the place of action because some delivery approaches would act only in the cytoplasm of the cells, while others are active in the cell nuclei. Recent technological advances facilitate a generation of even larger sequences of nucleic acids by chemically defined synthesis instead of using classical methods in molecular biology to produce recombinant nucleic acids by means of bacterial plasmids. The type of nucleic acids applied in the respective genomic therapy approach may also require normative considerations. In the following subsections, we aim to sketch the progress of those novel advanced molecular biology techniques that challenge the current regulatory framework. In order to relate these developments with some biological classifiers, such as mode and place of action, metaphor dimensions, or administration routes (see Table 1) we focus on new advances in (1) gene transfer by viral systems, (2) small non-coding RNAs and antisense oligonucleotide interference, (3) liposomal RNA delivery, and (4) targeted genome modification approaches.

**Table 1:** Somatic genomic therapies and their potential regulatory status

	Biomedical Classifiers			Regulatory Classifiers						
	Mode / Place of Action	Metaphoric Dimension	How is the active substance used in or administered to human beings	Does the active substance contain, or consist of, recombinant nucleic acid (rNA) – Y/N?	What is the genetic sequence (that is added, replaced, repaired, deleted)?	What takes place: regulation, reparation, replacement, addition, deletion of genetic sequence?	What is the therapeutic effect?	Is the therapeutic effect directly related to rNA or its product of expression?	What is, therefore, the regulatory status of the medicinal product?	Example^a^
Retro-/Lentiviral vectors	Integration of DNA / nucleus	(Permanent) additive gene transfer	In vivo / ex vivo	Yes	Recombinant viral RNA carrying a therapeutic gene	Addition of recombinant viral RNA	Substitution of missing gene enhancement of cells	Direct relation to therapeutic or enhancing protein expressed by the added gene	GTMP SCTMP	Encapsulated non-pseudotyped lentiviral vector encoding for CD19 CAR for in vivo targeting and transduction of T-cells (Treatment of CD19 + B-cell malignancy)
Adeno-(Associated) Viral Vectors	Non-integrating DNA as episomes / nucleus	(Long-term) additive gene transfer	In vivo / ex vivo	Yes	Recombinant viral DNA carrying a therapeutic gene	Addition of recombinant viral DNA	Substitution of missing gene enhancement of cells	Direct relation to therapeutic or enhancing protein expressed by the added gene	GTMP SCTMP	Replication incompetent gorilla adenovirus serotype 20 and modified vaccinia Ankara vectors encoding tumor-specific antigens mutant calreticulin and Janus Kinase 2 (Treatment of patients with myeloproliferative neoplasms)
(Liposomal) mRNA delivery	Direct mRNA-mediated expression / cytoplasm	(Short-term) additive gene transfer	In vivo / ex vivo	Yes/no depending on manufacture	mRNA	Addition of mRNA	Expression of therapeutically relevant protein	Direct relation to therapeutic protein expressed by the mRNA	Chemical medicinal product GTMP SCTMP	mRNA encoding the human glucose debranching enzyme (Treatment of glycogen storage disease III)
ncRNAs (ASOs, etc.)	RNA interference / cytoplasm	(Regulated) linear gene expression	In vivo	Yes/no depending on manufacture	ncRNA	Addition of ncRNA regulation of cellular mRNA	Activation or inhibition of expression of genetic sequence	Direct relation to ncRNA, e.g. binding cytoplasmatic mRNA	Chemical medicinal product GTMP	Synthetic antisense oligonucleotide capturing the mRNA transcript of the Transthyretin gene
AAV-mediated CRISPR-Cas delivery	Direct DNA modification / nucleus	Genome editing	In vivo / ex vivo	Yes	Recombinant viral DNA carrying a gene which expresses the CRISPR-Cas-Complex	Addition of recombinant viral DNA deletion or replacement of cellular DNA	Deletion or replacement of pathological genetic sequence	Direct relation to the product of expression, i.e. CRISPR-Cas complex	GTMP SCTMP	Analogue: E. coli containing a plasmid encoding a CRISPR-Cas system that targets three polyketide synthase (PKS) island genes (Prevention of disease progression in Familial Adenomatous Polyposis)
CRISPR-Cas Ribonucleoproteins	Direct DNA modification / nucleus	Genome editing	In vivo	SDN-1: no SDN-2; SDN-3: yes/no depending on manufacture	gRNA SDN-2; SDN-3: donor DNA	Addition of gRNA SDN-1: deletion of cellular DNA SDN-2/SDN-3: addition of donor DNA/replacement of cellular DNA	Deletion or replacement of pathological genetic sequence	No direct relation to rNA, i.e. gRNA SDN-2; SDN-3: direct relation to the product of expression of donor DNA	Biological medicinal product SDN-2; SDN-3: GTMP, if recombinant donor DNA	Nanoparticles formed by a cationic 4-armed polymer containing the gene editing components (CRISPR/Cas9 and the gRNAs) (Treatment of Recessive Dystrophic epidermolysis bullosa)
CRISPR-Cas Ribonucleoproteins	Direct DNA modification / nucleus	Genome editing	Ex vivo	SDN-1: No SDN-2; SDN-3: yes/no depending on manufacture	gRNA; SDN-2; SDN-3: donor DNA	Addition of gRNA SDN-1: deletion of cellular DNA SDN-2/SDN-3: addition of donor DNA/replacement of cellular DNA	Deletion or replacement of pathological genetic sequence	No direct relation to rNA, i.e. gRNA SDN-2; SDN-3: direct relation to the product of expression of donor DNA	SCTMP SDN-2; SDN-3: GTMP, if recombinant donor DNA	Genetically modified autologous CD34 + cell enriched population that contains haematopoietic stem and progenitor cells edited ex vivo by CRISPR/Cas9 at the erythroid-specific enhancer region of the BCL11A gene
CRISPRi/-a Ribonucleoproteins	Indirect DNA modification / nucleus	?	In vivo	No	gRNA	Addition of gRNA	Transcriptional activation/deactivation of therapeutic/pathological genetic sequence	No direct relation to rNA, i.e. gRNA	Biological Medicinal Product	Not yet available.
CRISPRi/-a Ribonucleoproteins	Indirect DNA modification / nucleus	?	Ex vivo	No	gRNA	Addition of gRNA	Transcriptional activation/deactivation of therapeutic/pathological genetic sequence	No direct relation to rNA, i.e. gRNA	SCTMP	Not yet available.

Examples are derived from scientific recommendations published by the EMA’s Committee for Advanced Therapies; cf. European Medicines Agency. https://www.ema.europa.eu/en/human-regulatory-overview/marketing-authorisation/advanced-therapies-marketing-authorisation/scientific-recommendations-classification-advanced-therapy-medicinal-products. Accessed 08 Oct 2024

### Gene transfer by viral systems

The first well-established viral vectors for gene therapy approaches were derived from murine leukaemia virus, which is a member of the sub-group (genus) Gammaretrovirus of the family Retroviridae. Such gamma-retroviral vectors consist of single-stranded RNA, which can be transcribed into double-stranded DNA by the enzyme reverse transcriptase, which is also encoded in the virus’s RNA genome. This double-stranded DNA can be integrated into the infected cell’s chromatin during the cell division phase by another virally encoded enzyme named integrase. Both features made gamma-retroviral vectors attractive tools for early additive gene therapy approaches, in particular in the field of hematologic gene defects, because intact copies of the disease-associated genes can be permanently introduced into the host cells’ genome to replace the missing gene function by an additive gene therapy approach. For this purpose, a DNA complementary to the mutated or missing gene’s mRNA (so-called cDNA) is generated by recombinant DNA technologies and implemented in the plasmid DNA that encodes the retroviral vector’s genome. This plasmid will be co-transfected with further helper plasmids to enable the production of infectious retroviral particles in suitable human cell lines. The relative ease of isolating hematopoietic stem/progenitor cells from bone marrow and the ability to culture them in vitro for retroviral transfection provided the basis for early gene therapy studies of hematologic genetic diseases. However, the integration pattern into the genome is biased towards genes that are actively transcribed in the transfected cells, which correlates with the risk of insertional mutagenesis by enhancing the activity of oncogenes such as cell cycle-related factors or by mutating putative tumour suppressor genes.

The in-depth research on human immunodeficiency virus (HIV), which belongs to the lentiviruses as a further genus of the family of Retroviridae, resulted in an advanced tool for gene transfer approaches. Such lentiviral vectors can even pass the nuclear membrane of non-dividing cells, which renders them suitable for gene therapy approaches in post-mitotic or quiescent non-dividing cells. Furthermore, the integration pattern of lentiviral vectors is more stochastic and, to a much lesser extent, biased towards actively transcribed host genes, which positively correlates with a safety profile preferable to that of gamma-retroviral vectors. In a modified version, lentiviral vectors consist of an altered integrase enzyme that does not integrate the nucleic acids cargo into the host cell’s DNA but allows for the delivery of lentiviral vector-encoded nucleic acids into the nucleus as free, so-called episomal, DNA molecules. Such integrase-deficient lentiviral vectors deliver DNA molecules to the nucleus of transfected cells, but the delivered DNA does not integrate into the host cell’s chromatin. Upon further division of such treated cells the episomal DNA molecules get diluted by being distributed to the respective daughter cells in each division cycle.

Another non-integrating vector system is based on adeno-associated viruses (AAVs). They belong to the family of parvoviruses and are dependent on helper viruses (e.g. adenoviruses) to maintain their life cycle. The size of the AAV genome only allows for the episomal delivery of rather small DNA sequences with a limit of roughly 5000 bases. Even if several natural AAV serotypes can infect human cells, no correlation to severe diseases has been described yet. Their viral capsid proteins can be easily modulated, which seems to be a versatile approach to reach tissue or cell type-specific delivery of the transgenic AAV cargo. The viral genome is encoded in a single-stranded DNA molecule, which reduces the chance of unintended integration into the host cell’s double-stranded DNA. Numerous clinical trials exploiting AAV-based delivery of transgenes are ongoing, with several products already approved by the FDA or EMA. However, during Phase 2 and Phase 3 clinical studies it became apparent that despite AAVs delivering single-stranded DNA, the processed double-stranded DNA intermediates might sporadically integrate into the host cells’ genome (Miesbach et al. 2023), and thus long-term follow-up observations were considered mandatory to identify putative oncogenic genomic alterations. So far, however, no event of oncogenic transformation upon AAV delivery has been confirmed despite several hundreds of treated patients. Recent scientific literature suggests the use of Sendai virus vectors in vaccine development or in gene therapy approaches, where short exposure of the expressed transgene might already cause a therapeutic benefit (Nakanishi et al. 2012). Sendai viruses belong to the family of Paramyxoviridae, which are enveloped single-stranded RNA viruses. Engineered Sendai virus derived vectors are well-established in biomedical research as robust delivery systems for expressing the reprogramming transgenes Oct4, Klf4, Sox2 and C-MYC in human cells for the generation of induced pluripotent stem cells (iPSCs). The key feature of this delivery system is that infected cells readily express the virally encoded single-stranded RNA in the cytoplasm, which confers a rapid and safe expression of the gene of interest. However, no advanced clinical study has been reported yet using Sendai virus vectors as a delivery tool.

All of these viral vector-based gene delivery systems may be considered as additive gene therapy approaches following the metaphoric understanding of DNA as “linear information”.

### Small non-coding RNAs and antisense oligonucleotide interference

The discovery of RNA interference, where small non-coding RNA molecules such as single hairpin RNAs (shRNAs) or small interfering RNAs (siRNAs) processed via intracellular protein complexes as well as antisense oligonucleotides (ASOs) can lead to mRNA degradation or suppress mRNA translation, has garnered a lot of attention in the last two decades. Meanwhile synthetic antisense oligonucleotides, which can be delivered to cells and suppress the mRNA translation of respective genes, are well-established. For example, Patisiran^®^, a siRNA targeting the Transthyretin protein causative for familial amyloid polyneuropathies or cardiomyopathies, was approved by the FDA and EMA, and this synthetic antisense oligonucleotide therapy product capturing the mRNA transcript of the Transthyretin gene is nowadays considered the standard therapy approach for these disorders.

Such therapeutic strategies still meet the concept of a linear understanding of genomic information where a gene sequence is transcribed into an mRNA, whose translation is subsequently blocked by the RNA interference pathway. However, other strategies involving microRNAs, which may simultaneously target multiple gene transcripts, need a more complex and dynamic understanding of the interplay of different genomic elements.

### Liposomal RNA delivery

Similar to above-mentioned small RNAs, the direct delivery of larger mRNA into target cells is a widely used approach in basic and translational science. The mRNA is a single-stranded RNA molecule that can be in vitro-transcribed using recombinant or synthetic DNA as a template for the reverse transcriptase enzyme or that can be directly synthesised as RNA oligonucleotide in a chemically defined manner. Packaging such mRNA into liposomal nanoparticles (LNPs), which can even be decorated with defined ligands that enable cell type-specific receptor-mediated uptake, is a recently established approach for genomic therapies (Toualbi et al. 2021). Such LNP-based delivery approaches were already broadly applied during the mRNA vaccine program of Moderna and BioNTech, where a modified, synthetic mRNA encoding different variants of the SARS-CoV-2 spike protein was expressed. Furthermore, LNP formulations are also widely studied to deliver components of precise genome editing tools, such as the CRISPR/Cas system.

### Targeted genome modification approaches

The CRISPR/Cas system for ‘genome editing’ approaches raises a lot of interest because of its relatively simple and modular concept. A short synthetically generated guideRNA (gRNA) complementary to the target DNA locus navigates the CRISPR/Cas complex to that spot, where the respective genome modification is intended. When CRISPR/Cas nucleases are applied, DNA double-strand breakes occur and get repaired via the error prone process of non-homologous end-joining (NHEJ). This approach can be applied to knock-down a disease-causing mutated gene. As an alternative approach, a modified Cas variant may be applied that only causes a single-strand DNA nick. Such Cas-nickase enzymes can be equipped with further functions acting at the targeted DNA locus, which may allow for a site-specific chemical modification of the respective nucleotide (‘base editing’). Here, a modified nuclease-deficient Cas protein directs an enzyme to a specific DNA locus, where a respective cytosine or adenosine deaminase can convert a cytosine into a thymine or an adenine into a guanine. In a ‘prime editing’ approach, a reverse transcriptase linked to the Cas protein transcribes the correcting DNA template from the co-delivered ‘prime-editing’-guide RNA (pegRNA) template. During the repair of the single-strand break, this corrected sequence gets integrated in the DNA and the corresponding opposite DNA strand gets subsequently corrected in a further step. Clearly, these CRISPR/Cas-based genome modification approaches refer to the metaphor “gene editing”.

A crucial step of all therapeutic CRISPR/Cas interventions is the appropriate delivery of the genome editing system to the respective cells in the organism. Here, the tissue- or cell type-specific delivery of the transgenic AAV cargo can be exploited for such novel ‘genome editing’ approaches. The AAV single-stranded DNA is delivered into the nucleus of the cell and encodes the sequence of the specific guide RNA together with the respective Cas variant (Cas nuclease, Cas ‘base editor’, Cas ‘prime editor’) in order to perform the intended gene modification (Schambach et al. 2024). In these circumstances, the additive genetic information of the Cas variant is delivered to the cells and the regulatory criteria of a gene therapy approach are also met. Alternatively, CRISPR/Cas nuclease proteins can be produced with modern biotechnology means and coupled with synthetic single guide RNAs to form Ribonucleoproteins (RNPs). Such RNPs can be packaged in liposomal nanoparticles for direct delivery into target cells (Schambach et al. 2024). Such RNPs get released in the cytosol and can enter the nucleus for gene disruption approaches by non-homologous end joining guided mismatch repair of the targeted genomic DNA locus. Such a strategy would not necessarily fall into the current definition of a gene therapy medicinal product (cf. 3.2), because no recombinant nucleic acids but only synthetic components are administered. Moreover, a CRISPR/Cas independent approach for targeted genome modification was recently developed, which does not consist of any nucleic acid, but only consists of protein content. Zinc-Finger nucleases (ZFNs) are proteins that cause sequence-specific DNA breaks and were previously also suggested to be used in targeted ‘genome editing’ approaches. Recently, modified ZFN-scaffolds were described, which include a deaminase allowing C: G-to-A: T ‘base editing’ in nuclear and mitochondrial DNA (Lim et al. 2022). Future applications of such ZFN-base ‘editors’ might use liposomal nanoparticle delivery technology to deliver such nucleic acid-free, protein-only tools for targeted ‘gene editing’ approaches. Nevertheless, both approaches, the RNP-mediated gene disruption and the ZFN-based ‘base editing’, modify the cells’ genetic sequences and are considered to be ‘genome editing’ applications.

Furthermore, the versatile CRISPR/Cas system can also be exploited for the activation or inhibition of the expression of a specific gene (Schambach et al. 2024). In such approaches, a nuclease-deficient Cas protein is coupled with strong transcriptional activator domains (such as VP64; CRISPRa-approach) or transcriptional repression elements (such as KRAB; CRISPRi-approach), which is navigated by the gRNA to the transcriptional start site of the respective gene. These CRISPR systems can be encoded and expressed by above-mentioned viral vectors or can be delivered by liposomal nanoparticle-mediated RNA transfer as suggested by a study using epigenetic editing of the PCSK9 gene in mice and cynomolgus monkeys to lower cholesterol levels in the blood (Tremblay et al. 2025). If the latter strategy was applied, the therapeutic application would neither meet the criteria for an (additive) gene therapy approach nor for a genetic sequence-modifying ‘genome editing’ approach.

### Interim conclusion

It can be summarised that the variety of methods utilised in contemporary genomic therapies indicates that the conventional term ‘gene therapy’, as well as the modern metaphorical term ‘genome editing’, are both insufficient to classify emerging novel techniques. The ability to control gene expression, e.g. using CRISPR systems, is no longer limited to ‘editing’; instead, other metaphors which are not confined to textual modifications such as “genetic switches” (Du et al. 2021) are also conceivable. To circumvent the problems associated with using a metaphorical term as a definiendum, we propose a more inclusive and neutral umbrella term: ‘somatic genomic therapies’ (SGTs). This term is intended to capture the broad array of techniques aimed at the precise alteration of gene expression at various molecular levels. Given the diversity of methods, the question arises whether regulatory frameworks are equipped to contain and facilitate their appropriate use. Considering the complexity of SGTs and the reliance of public discourse on insufficient and possibly misleading metaphors, considerable conceptual confusion at the basis of such legislation is to be expected. This hypothesis is exemplified in the following using the case study of the ATMP Regulation in the European Union (EU).

## The legislature’s metaphors and their limits

### EU regulatory framework

In Europe, gene and cell therapies are governed by Regulation (EC) No 1394/2007 on advanced therapy medicinal products (ATMP Regulation, EU 2007). This regulation distinguishes between three categories of ATMPs: gene therapy medicinal products (GTMPs), somatic cell therapy medicinal products (SCTMPs) and tissue engineered products (TEPs).^2^

The ATMP Regulation is a lex specialis. This means that, as a rule, general European medicinal products law applies to all life cycle stages of ATMPs, unless the ATMP Regulation stipulates special rules exclusively applicable to ATMPs (Schuessler-Lenz et al. 2023). Therefore, the classification of a medicinal product as an ATMP may have far-reaching regulatory implications within the EU.

For instance, ATMPs are subject to a centralised marketing authorisation procedure pursuant to Regulation (EC) No 726/2004 (EU 2004). This means that it is the European Commission which grants the authorisation for the placing on the market of ATMPs within the EU. Its decision is based on a scientific assessment conducted on the EU level by the European Medicines Agency (EMA) (EU 2004, Article 3(1) and Annex I No 1a, Article 10(2)). Within the EMA, the Committee for Advanced Therapies (CAT), a specialised scientific body composed of experts in fields relevant to advanced therapies, holds specific responsibility for evaluating ATMPs (ATMP Regulation, Article 8(3) and (4)). By contrast, medicinal products not classified as ATMPs can potentially be authorised via decentralised procedures on the national level (EU 2001, Article 6(1), 28(1)). Undergoing a decentralised authorisation procedure may be of relevance for small and medium-sized enterprises.

Furthermore, the classification as an ATMP entails distinct requirements under good manufacturing practice (GMP) standards: Part IV of the EU GMP Guidelines specifically addresses the manufacture of ATMPs, supplementing, or deviating from, the general principles set out in Parts I and II of said guidelines. In contrast, products that are not considered ATMPs are subject exclusively to these two general parts (cf. EU 2001, Article 46(f); ATMP Regulation, Article 5). As a result, ATMPs are subject to more stringent manufacturing requirements than conventional pharmaceuticals. This contributes to a substantial increase in manufacturing costs, infrastructure demands (e.g. clean rooms), and personnel qualification costs. Consequently, ATMP production is considerably more resource-intensive and expensive compared to traditional small-molecule or standard biological drug manufacturing.

On the other hand, in case of ATMPs, marketing authorisation fees may be reduced by up to 50% for SMEs (ATMP Regulation, Article 19(1)). In addition, SMEs are entitled to request scientific advice from the EMA, with fee reductions of up to 90% available for this service (ATMP Regulation, Article 16(2)). Hence, alongside the more stringent regulatory requirements intended to ensure safety and quality of ATMPs, the framework also provides targeted incentives for the development of ATMPs. However, these benefits are contingent upon the formal classification of the relevant medicinal product as an ATMP. In particular, the regulatory status as an ATMP, especially as a GTMP, is relevant for small research groups and biotech start-ups, as the ATMP regulatory framework aims at facilitating the translation of innovative therapeutic concepts into clinical application and eventual market access.

### The legislature’s concept of gene therapy and its limits as regards SGTs

SGTs do not only include classic gene therapy approaches, i.e. the insertion of therapeutically relevant genes via viral vectors. Rather, SGTs also enable the precise modification of the expression of genes at various molecular genomic levels (see Table 1), thereby extending the boundaries of conventional gene therapy. This raises the question of whether the ATMP definition, and more particularly the GTMP definition, captures such innovative approaches. For these legal definitions were adopted well before the seminal publication of the CRISPR/Cas technique in 2012 (Jinek et al. 2012) and the development of RNA technologies for medical purposes. Hence, certain novel forms of SGTs might fall outside the scope of the ATMP regulation, as the existing legal framework seems to operate on the classic approach to somatic gene therapies (supra 2.1) and its underlying overly simplistic metaphors.

The current legal definition of GTMP reads as follows: a GTMP “means a biological medicinal product which has the following characteristics:

- it contains an active substance which contains or consists of a recombinant nucleic acid used in or administered to human beings with a view to regulating, repairing, replacing, adding or deleting a genetic sequence;
- its therapeutic, prophylactic or diagnostic effect is related directly to the recombinant nucleic acid it contains, or to the product of genetic expression of this sequence”.

#### Linear thinking: the one-gene-one-protein paradigm

Accordingly, the GTMP definition requires that recombinant nucleic acids be used or administered to a human being for the purpose of, inter alia, adding a genetic sequence. As a result, under the GTMP definition, the classification of viral vectors inserting the therapeutically relevant genes into the genome of the patient, or the patient’s cells respectively, is relatively straightforward, as they represent the standard case, i.e. additive gene transfer. In case of in vivo^3^ approaches the active substance is the viral vector which is, as such, administered to the patient.^4^ Hence, the active substance is the virus, which ‘consists’ of the ‘recombinant nucleic acid’, i.e. the viral vector carrying the therapeutic gene, within the meaning of the GTMP definition. In contrast, in case of ex vivo^5^ approaches, the active substance is the cells to be transplanted into the patient.^6^ These cells have been genetically modified by using viral vectors beforehand. Hence, the active substances are the cells which, after transduction by the virus, ‘contain’ the ‘recombinant nucleic acid’ within the meaning of the GTMP definition. In both cases, the active substance is administered to the patient with a view to ‘add’ a genetic sequence, i.e. the therapeutic gene which has been combined with the viral vector. The added therapeutic gene will typically contain the information to encode a missing or function-enhancing protein to combat the patient’s disease. Consequently, the therapeutic effect will generally relate directly to the expression of the added genetic sequence.

The fact that classical gene transfer is so readily captured by the GTMP definition serves as an indication that the legislature at the time had in mind the metaphor, or the paradigm, of additive gene transfer as the conceptual foundation for the definition.

It is of note that the term ‘adding’ a genetic sequence does not necessarily require that the viral vector integrates into the genome, nor does it specify a certain place of action, e.g. the nucleus or the cytoplasm of the relevant cells. Hence, the plethora of viral vectors which do not integrate into the human genome, acting instead through episomal expression, would nonetheless fall within the scope of the GTMP definition. This would be the case, for example, with AAV or Sendai viruses. Above all, the functioning of GTMPs which ‘add’ a genetic sequence may also rely on other non-viral delivery systems, e.g. plasmids, bacteria, or fungi.^7^

Since integration into the cellular genome is not a constitutive element of the GTMP definition, one might assume that liposomal mRNA delivery could also fall within its scope. mRNA is a single-stranded copy of a gene, i.e. a nucleic acid, and encodes the genetic information for the synthesis of a particular protein by the cells’ ribosomes (NHGRI 2023b). For purposes of an in vivo approach, the mRNA may be administered to the patient through the injection of lipid nanoparticles (LNPs) enclosing the mRNA molecules.^8^ However, the relevant mRNA molecules are often chemically synthesised base-by-base.^9^ Chemically synthesised nucleic acids do not qualify as ‘recombinant nucleic acids’, as recombinant production requires the combination of nucleic acids from at least two different sources (cf. Stryjewska et al. 2013, p. 1076–1077; Mikić et al. 2023, p. 6; EMA 2015b, p. 99). Accordingly, such SGT products would form neither GTMPs (for lack of a ‘recombinant nucleic acid’) nor SCTMPs (for lack of somatic cells administered to the patient). Therefore, such mRNA-based SGT products would be beyond the scope of the current GTMP definition and, thus, outside ATMP regulation. If, by contrast, an exogenous gene is inserted into a DNA construct (forming together recombinant DNA) which is subsequently transcribed in vitro into mRNA, the resulting mRNA qualifies as recombinant mRNA and thus constitutes a ‘recombinant nucleic acid’. If this recombinant mRNA is administered to the patient in LNP formulations, a genetic sequence, namely, recombinant mRNA, i.e. the recombinant transcript of the recombinant DNA construct, is effectively added encoding the relevant therapeutic protein. Accordingly, this approach falls within the scope of the GTMP definition.

Accordingly, the existing GTMP definition does not cover synthetically manufactured mRNA, whereas mRNA transcribed from recombinant DNA can be subsumed under the GTMP definition. The European Commission and the Committee for Advanced Therapies (CAT) recognised this discrepancy between recombinant and chemically synthesised mRNA-products and agreed to solve these issues in the future (EMA 2020, p. 14; EMA 2021, p. 19).

Another RNA-based form of SGTs might make use of non-coding RNAs (ncRNAs), i.e. RNAs that do not produce proteins. Examples of such ncRNAs are ASOs, siRNAs, microRNAs, shRNAs. These ncRNAs can interfere with the mRNA’s function at the translational stage. Thus, they may be used to inhibit the production, e.g., of a disease-relevant protein the respective mRNA encodes for. ncRNAs are, generally, chemically synthesised and, therefore, respective SGT products cannot be classified as GTMPs (Guerriaud et al. 2022, p. 7). As a matter of interest, there are attempts to create recombinant ncRNAs (Yu et al. 2020, p. 890). Accordingly, respective medicinal products would contain an active substance which consists of recombinant ncRNA, i.e. of ‘recombinant nucleic acids’ within the meaning of the GTMP definition. The therapeutic effect, e.g., the inhibition of the expression of a pathological protein, would directly relate to the recombinant ncRNA. Therefore, de lege lata, the product would have to be classified as a GTMP (contrary to the ICH S12 guideline; cf. EMA 2023, p. 2). As the GTMP definition does not require that the therapeutic effect be mediated exclusively by the expression product of the transferred recombinant nucleic acid but also allows the recombinant nucleic acid itself to exert the therapeutic effect, it is possible for SGTs based on ncRNA to fall within its scope, even though they do not produce a protein. In this respect, the legislature appears to have shown a degree of foresight by seeking to capture a broader range of medicinal products beyond classical gene transfer.

#### Outgrowing the additive gene therapy concept: genome-editing

Departing from the traditional additive gene therapy concept of transferring genes, genome editing has introduced fundamentally new possibilities for functionally modulating gene expression only. Rather than relying on the addition of exogenous genetic sequences, these technologies enable targeted and precise alterations of endogenous genomic structures. For this reason, the classification of products based on CRISPR/Cas-based genome editing as GTMPs proves particularly challenging. While certain CRISPR/Cas applications resemble classical gene transfer, such as the insertion of exogenous genetic sequences, others entirely dispense with the notion of gene transfer, as is the case with pure gene knockouts. As a consequence, the limitations of the existing legal framework become particularly evident when examining the first generation of CRISPR/Cas applications, such as gene knockouts or the insertion of short nucleic acids (not forming genes in themselves) at predefined genomic loci, where the therapeutic effect results not from gene addition, but from targeted disruption or modification of endogenous genetic sequences.

The legal classification of CRISPR/Cas-based SGT approaches depends on the mode of delivery of the CRISPR/Cas complex, which consists of the Cas protein and the guide RNA (gRNA), into the cells (Liu et al. 2022, p. 333). Another important factor is the mode of operation (i.e. SDN-1, SDN-2 or SDN-3; Broothaerts et al. 2021, p. 24–41) of the particular CRISPR/Cas system.

In accordance with the original application of the CRISPR/Cas technology, the CRISPR/Cas complex is brought into the cells by using recombinant nucleic acids, e.g. recombinant AAVs which allow for the expression of both the Cas protein and the gRNA in the transduced cells. In this case, the CRISPR/Cas-based SGT product containing the recombinant AAV therefore may be classified as a GTMP.

However, the CRISPR/Cas complex can also be produced in vitro first as a ribonucleoprotein (RNP) and, then, be delivered as such into the cells through the use of lipid nanoparticles to be injected into the patient. This method could be used for an in vivo approach. In this case, the gRNA would be the only nucleic acid present in the SGT product administered to the patient. Since, however, the gRNA is chemically synthesised such an in vivo approach would not be captured by the GTMP definition and, thus, not be governed by ATMP regulation (Mourby et al. 2021, p. 980; Guerriaud et al. 2022, p. 7). Even if the gRNA was a recombinant nucleic acid, its *direct* relation to the therapeutic effect (as required by the GTMP definition) would be highly debatable, to say the least, because the gRNA only facilitates the site-specific genomic modification, while it is the nuclease, e.g. Cas9, a protein, that accomplishes the therapeutic effect through the double-strand break of the targeted genetic sequence.

Delivering the CRISPR/Cas complex into cells ex vivo, e.g. through electroporation, could lead to the SGT product’s classification as an SCTMP due to the cells’ substantial manipulation (cf. EMA 2015a, p. 11). Accordingly, in this case, the ATMP regulatory framework would still apply.

Nevertheless, one may ask the question whether the cells’ own genetic sequences, modified ex vivo, might be considered to be ‘recombinant nucleic acids’ within the meaning of the GTMP definition. From a traditional scientific perspective, ‘recombinant nucleic acids’ originate from a combination of nucleic acids of different living sources (Stryjewska et al. 2013, p. 1076–1077; Mikić et al. 2023, p. 6). Therefore, a mere disruption, or deletion, of the cells’ genetic sequences (SDN-1) would not fulfil this criterion. On the other hand, adding a particular piece of nucleic acid to the CRISPR/Cas induced process and joining this nucleic acid with the cells’ genetic material by their own repair mechanisms (SDN-2, SDN-3) could be considered as resulting in ‘recombinant nucleic acid’ within the cells. This would imply that the resultant CRISPR/Cas-modified cells might be classified as GTMPs as far as the resultant nucleic acid sequence is directly related to the therapeutic effect.

In summary, certain CRISPR/Cas-based approaches might be captured by the current GTMP definition and, therefore, governed by ATMP regulation, particularly those involving the addition of exogenous genes via genome-editing techniques, whereas others might not be, such as mere gene knockouts. The discrepancies can be attributed to the fact that the legal definition of GTMPs is conceptualised, at least primarily, along the lines of conventional gene therapies, which are characterized by additive gene transfer.

#### Beyond the gene therapy concept: epigenetics and the law’s struggle with conceptual change

SGTs which operate through epigenetic modes of action leave the nucleotide sequence of the relevant gene untouched and instead influence the expression levels of the gene, for instance, by modifying chemical markers on the gene’s DNA or altering specific regulatory proteins associated with the DNA. Hence, these SGTs typically do not consist of nucleic acids but rather of proteins and, thus, do not involve the introduction of genes, or any nucleic acids respectively, into cells. This simplifies the classification issue as such SGTs clearly fall outside of the scope of the GTMP definition. For instance, the definition of a GTMP does not encompass epigenetic SGTs such as CRISPRi or CRISPRa, provided these are delivered into human cells not via viral vectors but through lipid nanoparticles. In such configurations, the gRNA constitutes the sole nucleic acid component, whereas the Cas protein is catalytically inactive and no longer induces double-strand breaks in the genomic DNA. Instead, the mechanism of action consists in recruiting transcriptional repressors or activators to specific DNA sequences identified by the gRNA without adding any new genetic sequence. In such cases, the medicinal product does not contain any ‘recombinant nucleic acid’ (as, in particular, the gRNA is chemically synthesised), nor would its ex vivo application result in the generation of a ‘recombinant nucleic acid’ in the modified cells that are subsequently administered to the patient. Consequently, neither in vivo nor ex vivo applications of these agents acting on the epigenetic level would de lege lata fulfil the criteria for their classification as a GTMP. In ex vivo cases, the only remaining option would be classification as a SCTMP.

The fact that epigenetic modifications are beyond the scope of the legal GTMP definition may serve as further evidence that the legislature at the time was guided by additive gene therapy as the conceptual model.

### Framing regulation with metaphors: genome-editing in the language of proposed new regulation

A recent proposal by the European Commission seeks to amend the definition of GTMPs (EC 2023b, Article 4(1) Nr. 29) by introducing two distinct regulatory elements. According to the proposed definition, a GTMP is defined as:

‘a medicinal product, except vaccines against infectious diseases, that contains or consists of:

- a substance or a combination of substances intended to edit the host genome in a sequence-specific manner or that contain or consists of cells subjected to such modification; or.
- a recombinant or synthetic nucleic acid used in or administered to human beings with a view to regulating, replacing or adding a genetic sequence that mediates its effect by transcription or translation of the transferred genetic materials or that contain or consists of cells subjected to these modifications.’

#### Adapting to new standards, clinging to old concepts: genes as informational codes

This draft definition reveals, however, a continued adherence to the linear paradigm of molecular biology, particularly in point (b), where the regulatory classification is limited to nucleic acids whose function depends on being transcribed (DNA → RNA) or translated (mRNA → protein). The focus remains on the informational logic of genetic sequences as the actual therapeutic agents: substances are regulated as GTMPs insofar as they introduce readable and executable ‘code’ into the patient’s body. On the other hand, the proposal aims to ensure that such substances fall within the scope of regulation irrespective of their manufacturing method, be it recombinant production or chemical synthesis.

However, non-coding sequences, such as siRNAs, are excluded from the GTMP category despite also operating within the one-gene-one-protein logic, as they are neither transcribed nor translated in the patient’s cells. This inconsistency becomes particularly apparent when comparing mRNA and siRNA: although both act post-transcriptionally in the cytoplasm and do not integrate into the host genome, only the former qualifies as a GTMP under the proposed definition. Paradoxically, ncRNAs like siRNA may exert influence over transcription of endogenous genetic sequences in a manner that is even more fundamental than the influence of mRNA.

In addition, the draft introduces a new metaphor under point (a), implicitly drawing on the conceptual framework of ‘genome editing’. According to this provision, any substance intended to ‘edit’ specific sequences of the host genome qualifies as a GTMP. However, the legal text leaves crucial terms undefined: it remains unclear what constitutes ‘specific sequences’ and what the act of ‘editing’ entails. This ambiguity opens the door to considerable interpretive uncertainty, particularly in light of the fact that genome editing encompasses a wide range of modalities from precise base editing to large-scale chromosomal rearrangements. The legislature, thus, adopts a powerful yet vague technological metaphor without offering a corresponding operational definition, thereby exposing the regulatory system to conceptual overstretch and possible inconsistencies in enforcement.

#### Fragility of the ‘editing’ metaphor in the light of the collapse of genetic linearity

This can be exemplified by exploring the scope of the proposed GTMP definition which incorporates in point (a) the ‘editing’ metaphor. At first, the definition in point (a), explicitly refers to the ‘host genome’ rather than the transcriptome. The term ‘genome’ encompasses all DNA sequences located within the nucleus of a cell. Consequently, ‘editing the host genome’ under this provision must be understood as referring exclusively to alterations of DNA segments. Modifications to the transcriptome, which encompasses all RNA molecules of a cell, especially mRNA operating in the cell’s cytoplasm, are not captured by this definition.

This would only be different if any modulation of gene expression were to be interpreted as ‘editing’ the host genome in a ‘sequence-specific’ manner. Indeed, interventions at the epigenetic or transcriptomic level may indirectly affect the activity of the underlying genetic sequences. However, such an expansive interpretation would define the notion of ‘editing’ not as a substantial alteration of a genomic DNA sequence, but rather as any functional modulation of gene activity.

Such an interpretation would dramatically broaden the scope of point (a) of the proposed GTMP definition. This would be inconsistent with the internal logic of the provision. If followed through consistently, this interpretation would require classifying medicinal products containing ncRNA as GTMPs under point (a). Yet, these products are clearly excluded under point (b), which limits its scope to substances whose effect results from the transcription or translation of the transferred genetic material. This legislative choice reflects a clear intent to exclude ncRNA-based therapies from the GTMP category. An overly broad reading of point (a) would, therefore, contradict the structured limitation embedded in point (b).

Moreover, such reasoning would lead to absurd consequences. If any modulation of gene expression were deemed ‘editing’, then even the pharmacological degradation of a protein encoded by a human gene would have to be considered genetic modification. A narrower interpretation of ‘editing’ is therefore required which aligns with the teleological rationale underlying the definition. Since GTMPs are subject to heightened regulatory requirements, these should only apply where the intervention materially alters the genomic substrate itself.

Accordingly, the term ‘editing’ must be interpreted in a narrow sense: it refers exclusively to direct and substantive modifications of nucleotide sequences within the cell’s genome, as exemplified by the standard case of genome editing, i.e. genome editing by using site-directed nucleases. Epigenetic modifications – be it modifications of DNA markers or histones – and, in particular, CRISPRi/CRISPRa-based mechanisms, which merely guide transcriptional activators or repressors to predefined loci, are thus not captured by point (a) of the proposed GTMP definition. This exclusion persists even though certain epigenetic interventions – such as DNA methylation – entail genuine chemical modifications of the DNA molecule. These changes, however, do not alter the host genome in a sequence-specific manner itself. By adhering to the linear conception of genome editing – one that frames genes as linear codes composed of interchangeable ‘letters’ to be rewritten – the legislature limits the scope of regulation to interventions that modify the nucleotide sequence as such. The chosen metaphor of ‘editing’ thus privileges a view of the genome as a writable text, where only changes to the base sequence are legally meaningful. This approach, however, fails to capture epigenetic mechanisms that leave the informational content of the genome intact, yet profoundly alter its functional expression, particularly in terms of transcriptional accessibility and gene silencing.

### Interim conclusion

The current legal framework for ATMPs no longer adequately captures the latest developments in biomedical research and, by extension, potential future therapeutic approaches (see Table 1). The existing definition of GTMPs reveals that the legislature primarily had classical gene therapies via additive gene transfer in mind– namely, the insertion of a gene into a vector, implying a classical gene transfer. This conceptual framing, rooted in the technological standards at the time of drafting, no longer adequately captures the range of SGT approaches made possible by advances such as chemical synthesis of nucleic acids and the delivery of therapeutic molecules via lipid nanoparticles. It illustrates how a concept, paradigm or metaphor– established as the default model within a legal definition– can yield questionable regulatory outcomes when applied to novel technologies. Functionally equivalent therapeutic products are now subject to divergent regulatory treatment. For instance, CRISPR/Cas systems delivered via AAVs are classified differently from those delivered in LNP formulations (see Table 1). On the other hand, the legislature has made efforts to broaden the scope of the definition by including functional descriptors such as ‘to regulate’, ‘to repair’, or ‘to delete’ a genetic sequence alongside the classical ‘to add’ formulation. Nonetheless, due to the persistent influence of the virus-based additive gene transfer concept (‘recombinant nucleic acid’) regulatory practice defaults to scenarios involving the addition of a genetic sequence, thereby reinforcing the traditional logic of gene insertion.

The recent draft proposal by the European Commission intends to amend the GTMP definition to include ‘a substance or a combination of substances intended to edit the host genome in a sequence-specific manner’ (EC 2023b, Article 4(1) Nr. 29). The explicit reference to genome editing illustrates once more the legislature’s reliance on metaphors or guiding paradigms to delineate product categories. However, the increasing complexity and diversity of genomic therapies (see Table 1) reveals that it is far from self-evident what precisely constitutes an intervention that ‘edits’ the ‘host genome’. This ambiguity necessitates a broader philosophical and conceptual discourse to ensure that legal definitions are truly science-based and not misled by outdated or oversimplifying metaphors or paradigms.

## Philosophical perspective: an integrative postgenomic view of somatic genomic therapies

While there is a wealth of literature on the ethical challenges of genomic therapies, the philosophical aspects of classifying genomic therapies are an underexplored topic. A crucial worry in this respect is that an obsolete and misguiding understanding of the genome and related misconceptions about genomic interventions propagate not only in public discourse, but even reach codification in official legislation. As described in Sect. 3, one critical example for such a case is provided by the EU pharmaceutical package, currently under discussion, which includes a definition of genomic therapies in terms of “editing the host genome”. Notwithstanding the efficacy of such metaphors in science communication, precise definitions and appropriate classifications are crucial for establishing normative frameworks. The close examination of the metaphors commonly used to describe and classify genes, genomes, and genomic therapies then raises a number of philosophical challenges.

### The rise of information-related metaphors in biology

According to the biologist and historian Michel Morange (2020), the term ‘gene editing’ emerged as part of a language game that stems from the metaphor of the human genome as the ‘book of life’: The Human Genome Project (HGP), or so the story goes, made it possible to ‘read’ and ‘translate’ the ‘book of life’, so that biologists are now able to ‘rewrite’ and correct the ‘errors’ in it. These metaphors actively shape not only public perceptions of genomic research, but also molecular biology itself (Griffiths 2017). For example, the Somatic Cell Genome Editing Consortium of the US National Institutes of Health refers to the emerging techniques as “the move from reading to writing the human genome” (Saha et al. 2021). The genesis of metaphors such as reading, writing and editing can be traced back to what has been called ‘informational talk’ (Smith 2000) in biology, which emerged after the discovery of the double-helix structure of the DNA by Rosalind Franklin, James Watson and Francis Crick in the mid-20th century and the deciphering of the so-called ‘genetic code’ (Reynolds 2022). Following this, Crick formulated the ‘Central Dogma’ (and the related ‘sequence hypothesis’), which describes gene expression as a linear process: The flow of information in a cell proceeds unidirectional from nucleic acids (DNA and RNA) to nucleic acids and proteins (Crick 1970, p 562). The widespread acceptance of the Central Dogma gave rise to the ‘classical molecular gene concept’, which defines a gene as “a stretch of DNA that codes for one of the polypeptide chains that goes to make up a functional protein” (Stotz et al. 2004). This concept implies that a gene can be considered as a structural and functional unit within the genome, and that it has a direct influence on the phenotype through its effect on one or more of the structural elements that contribute to it (Stotz et al. 2004). In order to describe the results of the molecular advancements to the public, biologists have employed a series of metaphors derived from the fields of information theory, including the terms ‘blueprint’, ‘programme’ or ‘book’, which collectively convey the idea that a genome contains all of the information needed to build an organism (Griffiths 2006). In addition, informational vocabulary has had a notable impact on the field of genomics, with significant insights into the relationship between DNA structure and function emerging from the perspective of viewing DNA as a text comprising a four-letter alphabet that corresponds to the four nucleotide bases A, C, G, and T (adenine, cytosine, guanine, and thymine) (Barnes and Dupré 2008). Nevertheless, the practice of ‘informational talk’ has been the subject of criticism and debate within the field of the philosophy of biology, with the biologist and philosopher Sarkar (1996) going so far as to claim that “‘Information’ is little more than a metaphor that masquerades as a theoretical concept and leads to a misleading picture of the conceptual structure of molecular biology”. His chief contention is that ‘information’ and related concepts are imported from other fields (such as formal information theory) to biology not to elucidate the findings of genomics, but to “serve only as surrogates for nonexistent technical concepts”; in this way, informational talk occludes rather than addresses a serious lacuna in the conceptualization of the genome. Sarkar’s wholesale rejection of the term ‘information’ has proved controversial and may be criticised, among other things, for its overly rigid understanding of the transfer of terms between different disciplines (cf. Griffiths and Stotz 2013). However, one can certainly agree with philosopher Griffiths (2017) that the predominance of a certain class of informational metaphors has been partly responsible for the persistence of genetic determinism, i.e. the idea that the development of phenotypic traits is determined mainly by the presence of particular genes. So, while the metaphors of information need not be rejected as meaningless, terms such as ‘reading’, ‘writing’, and ‘editing’ are linked too closely to a classical, by now obsolete picture of the genome and therefore unable to reflect the advances made in molecular biology.

### The ‘postgenomic era’

In particular, the HGP has yielded a plethora of insights, prompting a re-evaluation and redefinition of foundational concepts such as the gene and the genome (Griffiths and Stotz 2013; Keller 2014; Guttinger 2020). One of these insights concerns the quantity of protein-encoding DNA sequences in the genome. These genes make up only 1.5% of the genome. The remaining sequences are non-coding and were originally referred to as functionless ‘junk’ DNA (Boland 2017). In 2003, the ENCODE (ENCyclopedia Of DNA Elements) Project Consortium (2012) was launched to “identify all functional elements in the human genome sequence”. Genomicists have since realised that these non-coding regions are not just junk DNA but play a crucial role in the timing and content of gene expression (Boland 2017). Another insight revealed by the HGP is that the flow of ‘genetic information’ in organisms is multidirectional or even circular (Cohen et al. 2016). The classical molecular gene establishes a one-to-one relationship between genes and proteins at the molecular level, which in turn are considered to establish a certain phenotype. However, the ongoing research has shown that there are also “one-to-many… [and] many-to-one correspondences between DNA segments and RNAs/polypeptides” (Meyer et al. 2013).

In response to these discoveries, researchers have proposed that we have entered the ‘postgenomic era’, defined as the period following the completion of the human genome sequence (Stevens and Richardson 2015). Despite the ongoing debate surrounding the term, a significant proportion of scholars regard postgenomics as a pivotal shift in biological thinking, one that transcends the limitations of genetic determinism and reductionism (Stevens and Richardson 2015). This is because, according to Griffiths and philosopher Karola Stotz (2004), the range of molecular actors has expanded significantly in postgenomics: “The genome is not merely a collection of genes, but houses diverse other functional elements. Genes no longer have a single function closely related to their structure, but respond in a flexible manner to signals from a massive regulatory architecture that is, increasingly, the real focus of research in ‘genetics.’” The most prominent illustration of this novel postgenomic knowledge is epigenetics, which, in the prevailing definitions, refers to the existence of mechanisms above the level of the gene capable of altering the expression of the genome without changing the DNA sequence itself (Lappé and Landecker 2015). According to the philosopher Marianne Boenink (2016), the fundamental philosophical premise of epigenetics is that molecules do not function in isolation, but only within the context of the cell, the organ, the body, and its environmental and social setting: “There is no essence in DNA or any other molecule that is necessarily realised in development, and which is juxtaposed to its environment (whatever that may mean). This implies that we should not conceive of bodies as passive and stable, something only acted upon by humans and their technologies.”

A further characteristic of this new postgenomic perspective includes also reframing of the material understanding of the genome. While the informatic model of the genome focuses on ‘coding DNA’, which is only a small component of complex genomes, and ignores most of the properties of the genome as a material entity, the ‘postgenomic’ genome is being reconsidered as a “concrete material object occupying space” (Dupré 2005). This ‘spatial perspective’ has its historical roots in the works of Hans Winkler (1920), who coined the term ‘genome’ for the first time in 1920 to indicate “the haploid number of chromosomes that represent the material basis of the systematic unit in association with the respective protoplasm”. Addressing not only space but also multidimensionality is the challenge that shapes postgenomic research (Mackenzie 2015). This is due to the fact that DNA within the nucleus is folded and compacted in an intricate manner, forming chromatin with a three-dimensional structure that influences genome function at several levels (Chen et al. 2023). For example, since genes and their regulatory elements can be spread over hundreds of kilobases, the spatial organisation of chromosomes allows them to be close together, enabling communication and further regulation of gene expression (Qu and Fang 2013). Consequently, alterations in the three-dimensional structure also contribute to the development of cancer and other diseases (Chen et al. 2023). It is therefore imperative to view the genome as a multidimensional research space (Larsen 2018).

As philosopher Keller (2015) observes, these findings have resulted in a paradigm shift in our understanding of the fundamental function of the genome, “transforming it from an executive suite of directorial instructions to an exquisitely sensitive and reactive system that enables cells to regulate gene expression in response to their immediate environment.” Drawing upon this postgenomic perspective of the genome, several authors have advocated for the diversification of metaphorical language with novel ones that more accurately reflect the advancements and ethical dilemmas posed by new technologies, as well as the complexity of genomic structures (Keller 2015; Perrault and O’Keefe. 2019). Such endeavours are driven by the objective of inventing metaphors that emphasise the complex interactions within the genome and between the genome and the environment. Hallam Stevens (2015) proposes that the most appropriate metaphor for the genomic space is that of a ‘biological network’. This is due to the fact that the genome would be similar to electronic or social networks, such as the Internet or Facebook, multiply connected in crisscross fashion and could be visualised through the same tools. As a demonstrative exemplar, Stevens employs the circular visualisation of the genome, generated by the software tool “Circos”^10^. This representation employs a circular configuration to illustrate genomic data, with lines intersecting at the centre to demonstrate various forms of connectivity among distinct regions of the genome. Furthermore, phenomena such as “cascades” in network science, where a change in one part of the network triggers a series of similar changes throughout the network, would allow for explanations as to how the networked genome would function: “In most cases it becomes clear that many genes, proteins, RNAs, or other molecules are involved in generating a phenotype. This might occur through cascades, or through other kinds of network interactions involving multiple elements. Using networks means becoming comfortable with such explanations, that is, with multiple elements acting together in ways that cannot always be completely unravelled, let alone predicted.” In this way, the effect of ‘activating’ a gene (for example, in the case of genomic modification) would become analogous to initiating a rumour within a social group or posting a video on the Internet: “the rumor may cascade through the network, or the video may “go viral,” but it is hard to predict the cases in which this will occur. We need more information not about the gene (or the video) but about the structure of the network through which it propagates.” This network-based approach would also support a novel causal explanation. In the networked genome, genes and proteins would retain their central role; however, they would no longer be determinative. The determinative element would be the way in which genes (and proteins) are *wired* together.

These interconnections and structure of the multidimensional genome also influence the understanding of how diseases occur. The deterministic thinking of classical medical genetics promoted the idea of a ‘one-gene-one-disease model’, in which genes are the root cause of disease, while other possible factors influencing the process are ignored (Boenink 2016). The discoveries made by the HPG led to two major classifications of diseases: monogenic diseases (involving one gene), and polygenic diseases (involving several genes). In addition to these, a third classification, multifactorial diseases (involving genes, the environment and their interaction), was also proposed (Boenink 2016). However, even monogenetic diseases have proven to be more complex than originally thought (Higgs and Wood 2008). For instance, in sickle cell anaemia - a disease that follows the classic Mendelian pattern of inheritance - a single point mutation in the adult haemoglobin (HbA) protein chain is present in all affected individuals. This mutation leads to the characteristic sickle-shaped deformation of erythrocytes. Despite this precisely defined mutation, the genotype does not allow any prediction of the patient’s phenotype: “some develop principally painful crises with or without bony infarcts; others are prone to hemolytic crises [...] while others are phenotypically normal, except for mild anaemia.” (Loscalzo et al. 2007). The aetiology of these heterogeneous clinical phenotypes appears to be multifactorial, involving the presence of additional disease-modifying genes and environmental influences that can interact to yield different phenotypes. Furthermore, in the fields of epigenetics and systems biology, disease processes are not regarded as law-like events, but rather as dynamic and evolving ‘entanglements’ that are essentially contingent (Boenink 2016). The metaphorical depictions of CRISPR/Cas as a tool for genome editing, akin to correcting spelling errors in a text, are indicative of both an antiquated perspective on the genome and the deterministic paradigm of classical medical genetics.

### Classifying techniques for modifying the multidimensional genome

The classification of genome-altering techniques necessitates consideration of the multidimensional and reactive genome, as novel technologies make the entire genome accessible and intervene in the complex ‘network of the genome’. In light of this research, it becomes evident that the term ‘genome editing’ is not an appropriate umbrella term for describing and classifying the range of new techniques: It equates the alteration of a gene with a change in function, even though the genome does not always exhibit such one-to-one correspondence. The metaphor also minimises the functional impact of the intervention; for example, the term ‘edits’ suggests minor, superficial adjustments, while those changes can have functionally meaningful changes. Additionally, they presuppose that modifications are limited to the primary structure, i.e., the DNA sequence, without accounting for possible effects on secondary or tertiary genomic characteristics. Finally, the metaphor of ‘editing’ implies a level of complete oversight and precision that does not correspond to the intricate and often unpredictable nature of genome-modifying technologies. The metaphor provokes these inapt inferences because it is rooted in a static, linear view of the genome, which does not reflect the genome as a dynamic, multidimensional space and its complex disease mechanisms.

Furthermore, the present discourse is characterised by substantial disagreement regarding the definitions of ‘gene therapy’, ‘genome editing’ and other emerging techniques, as well as the question of whether and how these terms should be distinguished from one another (Sharma et al. 2022). The fundamental premise of ‘conventional gene therapy’ is defined as “the replacement of defective genes with functional ones”. (Mohammadinejad et al. 2020). However, with the advent of novel techniques such as CRISPR/Cas, some argue that the definition of ‘gene therapy’ has changed (Landhuis 2021). This has led to efforts, such as those by Sherkow et al. (2018), to find a new definition of gene therapy that also encompasses emerging novel techniques such as CRISPR/Cas. However, genome-modifying techniques cannot be subsumed under the conventional term ‘gene therapy’ because they are not limited to genes. As described, the novel techniques also target non-coding regions of the genome, making ‘gene therapy’ no longer the appropriate umbrella term. Other researchers have taken a different approach, introducing novel categories of therapies. For example, they distinguish between the conventional approach of gene therapy on the one hand and ‘therapeutic genome editing’ (Doudna 2020; Cox et al. 2015) or ‘therapeutic gene writing’ (Pallarès-Masmitjà 2021) on the other hand. However, as we have argued in this section, the use of information-related metaphors such as ‘editing’ or ‘writing’ as overarching terms for classifying emerging techniques impedes an accurate understanding of genomic complexities and may mislead patients and the public. Even though every application of genome-modifying techniques requires a full assessment of potential risks, the use of such metaphors can compromise a clear understanding of the techniques involved and their potential dangers, since they promote an overly positive perception and downplay the associated risks. The classification is further complicated by the fact that ‘genetically modified cell therapy’, such as chimeric antigen receptor (CAR) T-cell therapy, has been questioned as to whether it can be categorised as gene therapy (Sharma et al. 2022; Cohnen et al. 2023). A similar issue arises with techniques that manipulate the epigenome. Given that some conceptualise the term ‘genome editing’ as excluding epigenetic alterations, this brings into focus another novel category of therapy, namely ‘epigenome editing’ (Enríquez 2021).

### An integrative concept of somatic genomic therapies

In light of these observations, we propose the umbrella term of ‘somatic genomic therapies’ to classify the described advanced molecular biology techniques. It is imperative to acknowledge that the objective of introducing this term is not to replace the ongoing endeavour to find a more appropriate metaphorical ‘landscape’ for the techniques and for the genome that are used by scientists to communicate with the public. Instead, we propose that, in order to facilitate the appropriate classification of the techniques, it is necessary to employ a non-metaphorical definition and umbrella term that are comprehensible to patients and the public without being misleading or overly simplistic.

Our proposal implies that a key criterion for determining whether a therapy is a *somatic genomic therapy* is *whether it uses advanced molecular biology techniques to add*,* precisely modify*,* or modulate a functional genomic element in a somatic cell for therapeutic purposes*. The tripartite structure of this proposal covers the wide range of possible medical interventions in the human genome as described in Sect. 2: From the insertion of functional gene copies into target cells to the modification of specific DNA sequences in the patient’s genome, and indeed any action that targets gene expression by means of manipulating processes such as transcription, translation or the epigenome, whether in vivo or ex vivo. It furthermore avoids building the editing metaphor into the definition of an SGT, thereby eschewing the problems identified above. As the target of an SGT we identify what the project ENCODE calls *functional genomic elements*, defined as “a discrete genome segment that encodes a defined product (for example, protein or non-coding RNA) or displays a reproducible biochemical signature (for example, protein binding, or a specific chromatin structure)” (The ENCODE Project Consortium 2012). As compared to a more traditional notion of a gene, the concept of a functional genomic element is therefore better equipped to accommodate two fundamental facts of contemporary molecular biology: First, research on genome function is not merely conducted on isolated DNA sequences; rather, it is carried out in the context of the cell, i.e. in different cell types and activity states (Germain et al. 2014). Second, “common biochemical or biophysical events that invariably attend certain types of noncoding functional elements” are recognised (Stamatoyannopoulos 2012). The ENCODE approach therefore corresponds better to an appropriately postgenomic understanding of the genome that extends beyond genes as protein-coding regions to consider non-coding transcripts and functional genomic elements involved in the *temporal and spatial* regulation of gene expression. Moreover, identifying functional genomic *elements*, rather than the *host genome*, as the target of an SGT sidesteps the issue of the lack of an uncontroversial definition of the term ‘genome’ and the ambiguity that arises from the absence of clearly defined boundaries. Lastly, the proposal is forward-thinking and open to future research developments in that it accounts for functional genomic elements that may yet prove to have an impact on biomedical research, even if not yet identified as disease relevant (The ENCODE Project Consortium 2012).

Our concept of SGTs is therefore integrative as it aims to encompass existing and future advanced molecular biology techniques that can precisely target existing and yet-to-be-discovered functional genomic elements associated with diseases. These techniques encompass conventional gene transfer as well as postgenomic approaches such as epigenetic manipulation. The need for a forward-looking and integrative concept of SGTs is an urgent issue, as there are currently a number of regulatory uncertainties and inconsistencies in various legislations regarding SGTs, due to a lack of flexibility in the respective regulatory frameworks to respond to the rapid progress in genomic research and the development of genomic therapies. The field of functional genomics is an open-ended discipline, with the potential to provide insight into numerous unanswered questions pertaining to the genome, including its dynamics and effects on health and disease (Banerjee 2024).

## Conclusion

The classification of genomic therapies is a complex issue due to the rapid technological progress, the inertia of legislation lagging behind the state of science and technology, and the philosophical issues surrounding the spatial and multidimensional understanding of the genome. One source of confusion arises from the fact that the definitions of the genome as well as those of emerging genomic technologies are based on informational metaphors that have been superseded by developments in the field. The lack of discussion about classifications and definitions leads to uncertainty about what exactly genomic therapies are and thus how they are to be evaluated in a normative framework. Here, we have outlined an integrative approach of SGTs in order to overcome the current confusion and bring in the philosophical discourse around the genome. This approach requires further elaboration and is unlikely to resolve all debates. However, it aims to stimulate further discussion and refocus the debate on the key issues: to convey a public understanding of the genome and its therapeutic modification according to actual research results and to lay the conceptual groundwork for the development of normative guidelines and regulatory frameworks to ensure that SGTs can be safely and rapidly translated into the clinic.

## Abbreviations

ASOs: Antisense Oligonucleotides
ATMP: Advanced Therapy Medicinal Product
CAT: Committee for Advanced Therapies
EMA: European Medicines Agency
gRNA: guide RNA
GTMP: Gene Therapy Medicinal Product
ICH: International Council for Harmonisation of Technical Requirements for Pharmaceuticals for Human Use
ncRNA: non-coding RNA
SCTMP: Somatic Cell Therapy Medicinal Product
SGT: Somatic Genomic Therapy
shRNA: single-hairpin RNA
siRNA: small interfering RNA
TEP: Tissue Engineered Product

## Footnotes

1 In their study, Sherkow et al. delineate the issue of demarcation between genomic and non-genomic therapies as follows: “Since Watson and Crick’s discovery of the molecular structure of DNA, the ‘Holy Grail’ of molecular biology has been the ability to precisely edit the genome of in living tissue. The functional idea behind this aspiration is that by replacing some of a body’s genetic material, researchers and clinicians can effectively reprogram genetic illnesses. But underlying this goal lies a deep-philosophical line-drawing problem: all therapies, in some sense, affect a patient’s genes, whether it’s through altering transcription, regulating translation, or even modifying the epigenome. A relaxing vacation - sun, surf, and sangria - arguably does more to regulate gene expression than many ‘precision’ therapies” (Sherkow et al. 2018).

2 Article 2(1)(a) of the ATMP Regulation (EU 2007).

3 An in vivo gene therapy means that the genetic modification of cells intended to exert a therapeutic effect occurs only within the patient’s body (Malech et al. 2022).

4 Cf. No 3.2.1.1. of Part IV of Annex I to Directive 2001/83/EC (EU 2001).

5 An ex vivo gene therapy means that the respective genetic modification takes place outside the patient’s body through a genetic alteration of cells in vitro which are subsequently transplanted into the patient (Malech et al. 2022).

6 Cf. No 3.2.1.2. of Part IV of Annex I to Directive 2001/83/EC (EU 2001).

7 Cf. No 3.2.1.1. (2), 3.2.1.2. (2) of Part IV of Annex I to Directive 2001/83/EC: “genetically modified microorganism(s)” (EU 2001).

8 Cf. No 3.2.1.1. (2) of Part IV of Annex I to Directive 2001/83/EC: “nucleic acid sequence(s)” (EU NHGRI).

9 Longer mRNA molecules may need to be biotechnologically engineered. However, with further scientific progress, longer mRNA chains might be chemically synthesised, as well (Quabius et al. 2015, p. 230; Sahin et al. 2014, p. 7).

10 See https://circos.ca/intro/genomic_data/ (Krzywinksi et al. 2009).
